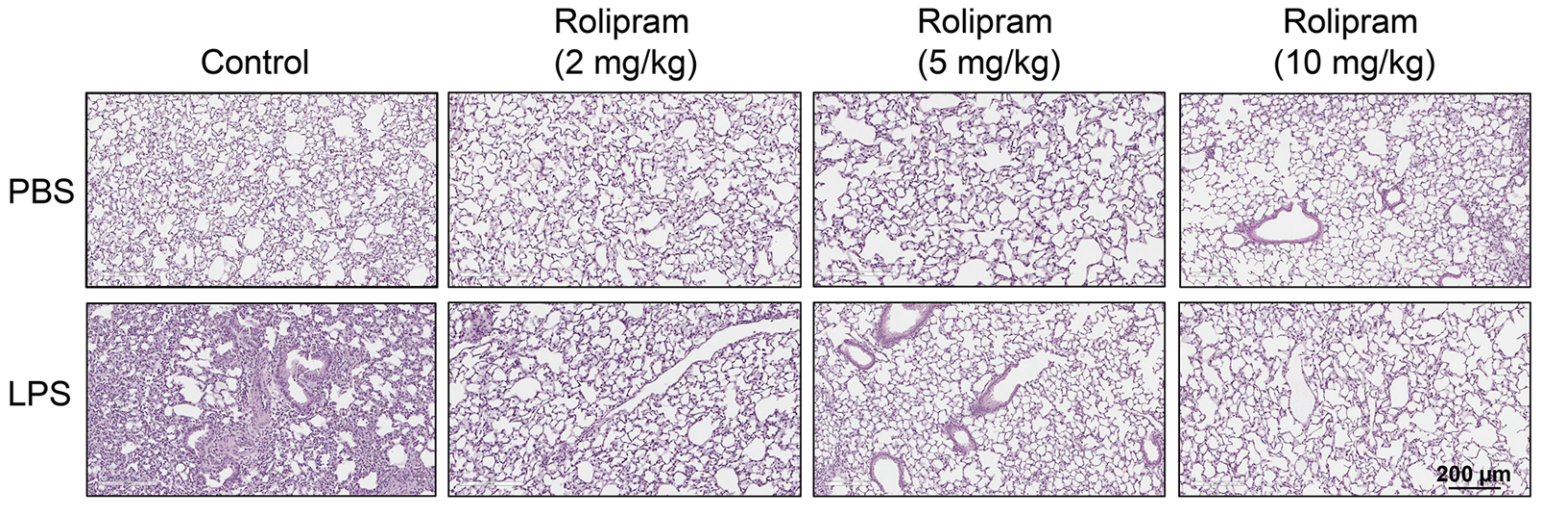# IL-1β suppression of *VE-cadherin*﻿﻿ transcription underlies sepsis-induced inflammatory lung injury

**DOI:** 10.1172/JCI169500

**Published:** 2023-03-01

**Authors:** Shiqin Xiong, Zhigang Hong, Long Shuang Huang, Yoshikazu Tsukasaki, Saroj Nepal, Anke Di, Ming Zhong, Wei Wu, Zhiming Ye, Xiaopei Gao, Gadiparthi N. Rao, Dolly Mehta, Jalees Rehman, Asrar B. Malik

Original citation: *J Clin Invest*. 2020;130(7):3684–3698. https://doi.org/10.1172/JCI136908

Citation for this corrigendum: *J Clin Invest*. 2023;133(5):e169500. https://doi.org/10.1172/JCI169500

In the original version of Figure 4D, the representative lung histology sections of control and LPS-exposed animals, specifically the Rolipram (5 mg/kg)/PBS, Rolipram (5 mg/kg)/LPS, Rolipram (10 mg/kg)/PBS, and Rolipram (10 mg/kg)/LPS samples, inadvertently showed overlapping images from the same slide due to an error in figure assembly. The correct figure is shown below.

The authors regret the error.

## Figures and Tables

**Figure:**